# “CAPA in Progress”: A New Real-Life Approach for the Management of Critically Ill COVID-19 Patients

**DOI:** 10.3390/biomedicines10071683

**Published:** 2022-07-13

**Authors:** Nieves Carbonell, María Jesús Alcaráz, Ainhoa Serrano-Lázaro, María Rodríguez-Gimillo, David Sánchez Ramos, Francisco Ros, Josep Ferrer, María Luisa Blasco, David Navarro, María Ángeles Clari

**Affiliations:** 1 Medical Intensive Care Unit, Clinic University Hospital, INCLIVA Health Research Institute, 46010 Valencia, Spain; aserranolazaro@gmail.com (A.S.-L.); mariarodriguezgimillo@gmail.com (M.R.-G.); francisco.rosv@gmail.com (F.R.); mablas13@gmail.com (M.L.B.); 2 Microbiology Service, Clinic University Hospital, INCLIVA Health Research Institute, 46010 Valencia, Spain; alcaraz_marsor@gva.es (M.J.A.); david.sanchezramos@gmail.com (D.S.R.); ferrer.jmf@gmail.com (J.F.); david.navarro@uv.es (D.N.); maclapons@hotmail.com (M.Á.C.)

**Keywords:** COVID-19, critically ill patients, CAPA, “CAPA in progress”, galactomannan, *Aspergillus* spp., tracheal aspirate, acute respiratory distress syndrome

## Abstract

(1) Background: COVID-19-associated pulmonary aspergillosis (CAPA) has worsened the prognosis of patients with pneumonia and acute respiratory distress syndrome admitted to the intensive care unit (ICU). The lack of specific diagnosis criteria is an obstacle to the timely initiation of appropriate antifungal therapy. Tracheal aspirate (TA) has been employed under special pandemic conditions. Galactomannan (GM) antigens are released during active fungal growth. (2) Methods: We proposed the term “CAPA in progress” (CAPA-IP) for diagnosis at an earlier stage by GM testing on TA in a specific population admitted to ICU presenting with clinical deterioration. A GM threshold ≥0.5 was set as the mycological inclusion criterion. This was followed by a pre-emptive short-course antifungal. (3) Results: We prospectively enrolled 200 ICU patients with COVID-19. Of these, 164 patients (82%) initially required invasive mechanical ventilation and GM was tested in TA in 93 patients. A subset of 19 patients (11.5%) fulfilled the CAPA-IP criteria at a median of 9 days after ICU admittance. The median GM value was 3.25 ± 2.82. CAPA-IP cases showed significantly higher ICU mortality [52.6% (10/19) vs. 34.5% (50/145), *p* = 0.036], as well as a much longer median ICU stay than those with a normal GM index [27 (7–64) vs. 11 (9–81) days, *p* = 0.008]. All cases were treated with a pre-emptive systemic antifungal for a median time of 19 (3–39) days. (4) Conclusions: CAPA-IP highlights a new real-life early approach in the field of fungal stewardship in ICU programs.

## 1. Introduction

Invasive pulmonary aspergillosis (IPA), a clinical condition affecting immunocompromised patients, has been well known since the EORTC/MSG group-developed consensus of 2008. However, evidence related to non-neutropenic critically ill patients emerged much later. In 2012, the AspICU study group proposed an algorithm with the potential to rule out IPA in this population [1]. Accordingly, an observational multicenter study including 563 intensive care unit (ICU) patients demonstrated a putative IPA incidence of 36%, with significantly higher mortality than from respiratory fungal colonization alone (67% vs. 38%) [2].

During the current pandemic, different studies have contributed to shaping and highlighting the role of respiratory superinfection by *Aspergillus* spp. in a severe course of SARS-CoV-2 infection. Although it has peculiarities differentiating it from influenza-associated pulmonary aspergillosis (IAPA) [3], it also apparently casts a shadow over the prognosis of COVID-19 pneumonia patients with associated acute respiratory distress syndrome (ARDS). In daily clinical practice, however, the lack of specific criteria for diagnosing IPA in ICU patients hampers the timely initiation of appropriate antifungal therapy, and as such may compromise the odds of survival. Because of this, in December 2020, a consensus statement for defining and managing a new clinical entity referred to as COVID-19-associated pulmonary aspergillosis (CAPA) was published [4]. Authors underlined the importance of bronchoalveolar lavage (BAL) as the validated sample of choice to define patients with “probable” CAPA. However, considering the difficulties in collecting low respiratory samples entailed by the pandemic, samples obtained from non-bronchoscopic lavage were proposed as an alternative to BAL to diagnose “possible” CAPA.

Tracheal aspirate (TA) samples continued to be considered unhelpful because they were not validated for biomarker detection, primarily due to the risk of diagnosis by a positive culture result and of overtreating upper airway colonization as if it were IPA. However, it should be noted that during the pandemic, observational studies assessed the screening role of TA for probable CAPA in ICU patients using *Aspergillus* spp. culture or polymerase chain reaction (PCR) [5], as well as by galactomannan (GM) assay [6]. Moreover, in real-life studies of CAPA with ARDS in ICU patients, a non-depreciable percentage of diagnostic cases were established using TA instead of BAL samples [6,7,8,9,10].

It has been proposed that, under certain host factors (critically ill patients, acute respiratory virus infection, and ARDS), poor clearance of conidia enables their germination into hyphal morphotypes. GM detection is also highly indicative of IPA, as this antigen is released during active fungal growth [8]. Thus, based on disease pathophysiology, we proposed the term “CAPA in progress” (CAPA-IP) as the rationale to carry out an active aspergillosis search by GM testing on TA in a specific ICU population, as well as contribute to improving fungal stewardship programs.

## 2. Materials and Methods

### 2.1. Microbiological Data

The presence of SARS-CoV-2 in respiratory samples was confirmed by reverse transcription PCR (RT-PCR) (TaqPath COVID-19 Combo Kit (Thermo Fisher Scientific, MS, USA)). The detection of 1,3-β-d-glucan (BDG) in serum was performed with the Wako β-glucan test (Fujifilm Wako Pure Chemical Corporation). GM testing was performed using Platelia™ Aspergillus (Bio-Rad Laboratories). For detection of Aspergillus DNA, we used an Aspergillus PCR assay [AspID (OLM Diagnostics, Newcastle, UK)]. GM, Aspergillus DNA in TA and serum-BDG were analyzed in cases of suspected fungal infection.

Culturing of respiratory samples was performed upon clinical request on Sabouraud agar with chloramphenicol (Becton-Dickinson, USA). To avoid the risk of environmental contamination of the TA, it was initially obtained through the Halyard Turbo-Cleaning Closed Suction System as the one described for possible CAPA definition [11]. Moreover, all samples were worked in a Class II biosecurity hood and Sabouraud chloramphenicol media sealed with Parafilm M^®^ paper (Bemis Company, Inc., Neenah, WI, USA). Species identification was performed by MALDI-TOF MS (Bruker Daltonik GmbH, Bremen, Germany). Score values ≥1.8 were accepted as reliable identification. The study of sensitivity to antifungals was carried out by microdilution in broth (SensititreTM, Thermo Scientific, UK) following the European Committee on Antimicrobial Susceptibility Testing (EUCAST) breakpoint tables for interpretation of MICs for antifungal agents’ criteria, version 10.0, 2020.

### 2.2. Case Definition of CAPA-IP

In this prospective observational study, we analyzed consecutive patients admitted to the ICU in the Clinic University Hospital (CUH), Valencia, Spain, between March 2020 and January 2022 with a diagnosis of COVID-19 pneumonia and secondary ARDS. COVID-19 was confirmed with a SARS-CoV-2-positive nasopharyngeal swab by RT-PCR. 

In patients presenting with clinical deterioration over the disease course suggestive of respiratory superinfection (fever, higher acute phase reactants (APR), and greater hypoxemia or changes in respiratory secretions) regardless of the presence or not of radiological changes, a new TA was obtained through the same Halyard Turbo-Cleaning Closed Suction System as the one described for possible CAPA definition, but without using 40 mL saline [11].

For diagnosis of the stage named CAPA-IP (not yet fully established angioinvasion), we set a GM threshold ≥0.5 as the mycological inclusion criterion by extrapolating the validated values from BAL samples [12]. In our hospital center, GM results are provided by the microbiology laboratory in a few hours. An associated positive result for *Aspergillus* spp. PCR or subsequent growth in culture for *Aspergillus* spp. in the TA sample strengthened the case for inclusion, as did serum positivity BDG with 7 pg/mL as the cutoff for positivity, corroborating an invasive fungal disease (IFD). Subsequently, weekly GM tests were obtained from TA samples until the patient was either discharged from ICU or died.

### 2.3. Statistical Analysis

Frequency comparisons for categorical variables were performed using Fisher’s exact test. A Student’s t-test was used to compare quantitative data with normal distribution, and the Mann–Whitney U test for non-normally distributed data. Values were expressed as mean ± standard deviation (SD), median (range), or a percentage when appropriate; *p* value < 0.05 was considered statistically significant. The analyses were performed using SPSS version 20.0 (SPSS, Chicago, IL, USA). 

The current study was approved by the Research Ethics Committee of CUH INCLIVA (July 2020). All experiments were performed in accordance with corresponding local guidelines and regulations. Informed consent was obtained from all participants, either at the time of ICU admission or during short follow-up.

## 3. Results

We prospectively enrolled 200 consecutive patients admitted to our sixteen-bed medical ICU with a diagnosis of COVID-19 pneumonia over an almost two-year period. In the overall study population, the median age was 66 years (range: 21–82), 66% were male, with a mean APACHE II score of 15 ± 6 and SOFA score of 5 ± 3 on the first ICU Day. Regarding clinical characteristics that are widely considered as host-related risk factors for aspergillosis, we observed that 5 (2.5%) out of the whole population included suffered from a hematological disease, 9 (4.5%) were under immunosuppressive drugs at ICU admittance (5 patients because of solid neoplasia and 4 patients because of autoimmune disease), 9 (4.5%) were diagnosed with chronic obstructive pulmonary disease (COPD), and 2 (1%) with cirrhosis. None of them had solid organ transplantation or VIH infection. In the first 48 h following ICU admittance, 164 patients (82%) required invasive mechanical ventilation (IMV), 71% norepinephrine infusion, and 8% renal replacement therapy. Most were initially treated with corticosteroids (74%). During their ICU stay, 28% of the patients developed ICU-acquired respiratory infections (including ventilator-associated tracheobronchitis and pneumonia). The overall ICU mortality in the total population was 30.5%.

**Figure 1:**
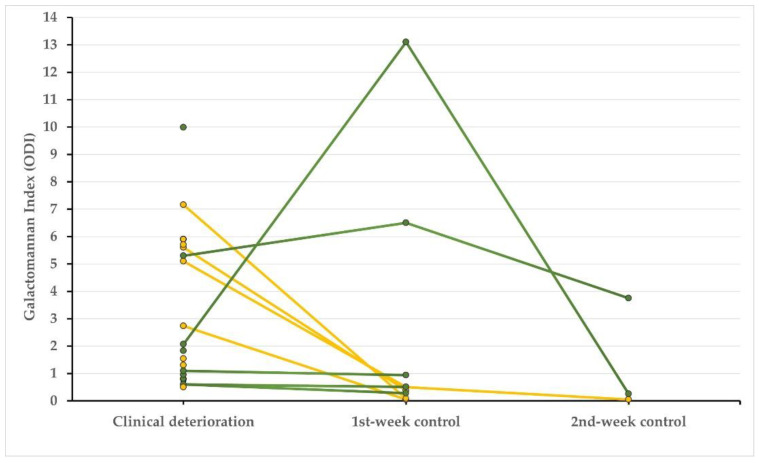
Galactomannan values in consecutive tracheal aspirate samples during ICU stay in patients diagnosed with COVID-19 pneumonia and CAPA in progress. Green lines and symbols denote patients who died, and those who survived are indicated in yellow.

GM was tested in TA because of new-onset clinical deterioration in 93 out of 164 patients requiring IMV, with a positive result in 20% of samples. Therefore, a subset of 19 (11.5%) critically ill ventilated patients with COVID-19 pneumonia and ARDS fulfilled the CAPA-IP criteria. Among these patients, two of them (10.5%) were under treatment with immunosuppressive drugs at ICU admittance, and one (5%) was diagnosed with mild COPD. All cases had received corticosteroids and 47% also received tocilizumab. The clinical deterioration episode occurred at a median of 9 (2–19) days after ICU admittance. The median GM value in the TA sample obtained at this time was 3.25 ± 2.82 ODI. Five out of nineteen cases had been treated with piperacillin/tazobactam during the previous week; however, afterwards, three of them tested positive for fungal culture, potentially resulting in 10% false-positive GM assay results. The kinetics of GM values across the following weeks of ICU stay are shown in Figure 1. Fungal culture tested positive in 7 out of 19 (37%) patients with CAPA-IP (3 *A. fumigatus*, 3 *A. terreus,* and 1 *A. flavus*). The serum BDG was positive in five cases (26%). Microbiological data are shown in Table 1.

Moreover, the subset of patients with COVID-19 pneumonia and ARDS under IMV who developed CAPA-IP showed significantly higher ICU mortality than those invasively ventilated patients who did not [52.6% (10/19) vs. 34.5% (50/145), *p* = 0.036]. Furthermore, ICU stays were much longer than in those with a normal GM index [27 (7–64) vs. 11 (9–81) days, *p* = 0.008].

All cases (except for two who died on the day of diagnosis) were treated with pre-emptive systemic antifungal therapy as soon as the GM assay results were available (11 with isavuconazole, 5 with voriconazole, and 1 with an echinocandin, according to physician criteria), and showed a similar mortality rate as when managed with different azoles (54.5% vs. 40%, respectively, *p* = 0.59) (Table 1). Liposomal amphotericin B was employed as salvage treatment in two patients, both of whom unfortunately died. The median antifungal treatment time was 19 (3–39) days. In the surviving cases, it was withdrawn in accordance with physician criteria based on clinical stabilization, GM value normalization, and gasometrical and radiological improvement. GM was determined in a BAL sample, when possible (mainly after ICU discharge to the pneumology ward), to inform decision making in follow-up.

## 4. Discussion

Despite efforts to unify criteria regarding IPA definitions, the idiosyncrasy of each situation, such as in the current pandemic, and between-center variations in microbiologic laboratory test availability and ICU procedures and training, make it difficult to generalize and protocolize CAPA diagnostic measures and management. Nonetheless, it continues to be a worrisome clinical entity with a high associated mortality rate. Taking these two aspects into account, our approach was to carry out an active aspergillosis search in susceptible hosts, which were defined as critically ill patients with SARS-CoV-2 virus pneumonia and ARDS requiring IMV, most receiving immunosuppressive treatment in our unit. New-onset clinical deterioration and mycological evidence were subsequently added to complete the search. Given that GM antigens are released during active fungal growth, we decided to analyze the diagnostic value of this biomarker on TA samples, an easy and well-known technique not yet validated for biomarker detection. 

**Figure 2:**
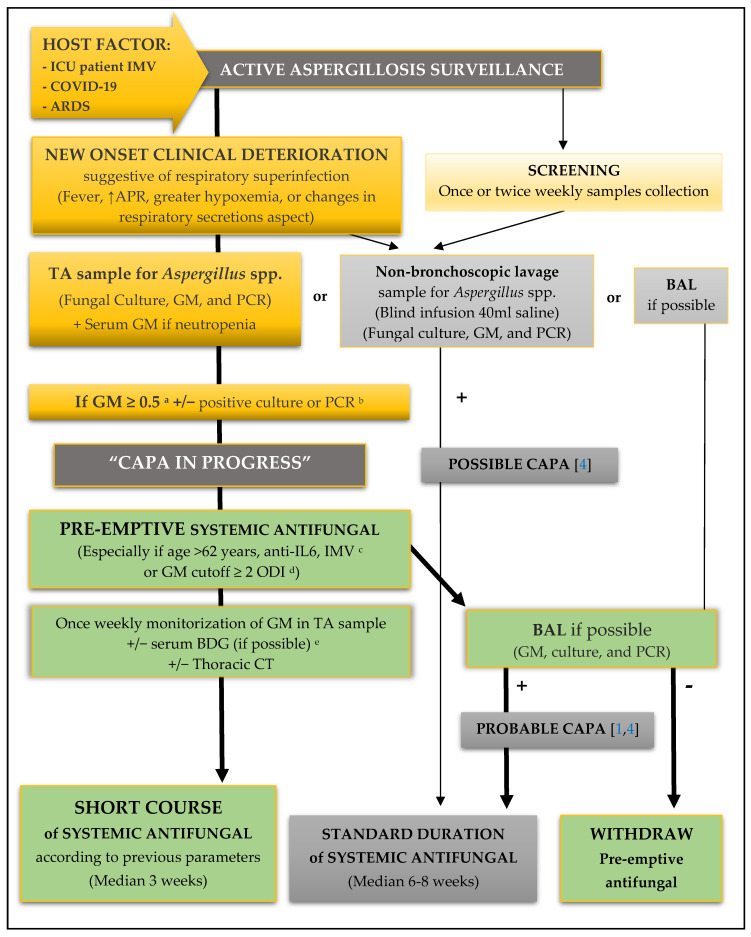
Algorithm for diagnosis and management of “CAPA in progress” in patients admitted to ICU diagnosed with COVID-19 pneumonia and ARDS requiring IMV. Possible approaches depend on hospital characteristics. The protocol followed in our study is shown on the left side while the previously described screening protocols on the right one [5,6,10]. Key aspects supporting our protocol in the algorithm are: ^a^ GM from TA is a subrogate of the emerging invasive process as the antigen is released during active fungal growth, providing the pathophysiologic basis of the CAPA-IP concept [8]. ^b^ Detection of *Aspergillus* spp. in respiratory samples (TA) by culture or PCR is associated with mortality [10]. ^c^ These three factors were independently associated with proven, probable, or possible CAPA in two large cohorts of critically ill patients diagnosed with COVID-19 pneumonia and ARDS [10,13]. ^d^ Diagnostic performance of GM in TA is improved using a cutoff value of 2 ODI, with a negative predictive value (NPV) of over 90% for CAPA probable diagnosis [6]. ^e^ Mean serum BDG value in patients with proven and probable invasive fungal disease is significantly higher than in those with fungal colonization, with an NPV of 83% [14]. ICU: intensive care unit; IMV: invasive mechanical ventilation; ARDS: acute respiratory distress syndrome; APR: acute phase reactants; TA: tracheal aspirate; GM: galactomannan; PCR: polymerase chain reaction; ODI: optical density index; BDG: (1-3)-β-d glucan; CT: computer tomography. Ref. [4] and refs. [1,4] refer to respective bibliographic references.

The optimal diagnostic workflow for CAPA is currently unknown, but since the 2020 ECMM/ISHAM consensus, new high-quality evidence has been published. We thus established the concept of CAPA-IP as a new subset in the CAPA classification, which indicates an earlier stage in the IPA pathophysiology process during *Aspergillus* spp. conidial germination and increased potential invasion. Accordingly, we also propose a new algorithm for the diagnosis and management of this stage, which includes a pre-emptive strategy with a short-course systemic antifungal (Figure 2).

The current study therefore highlights some novel aspects in the field of coinfection between SARS-CoV-2 and *Aspergillus* spp. First, it provides a new GM cutoff value on studied, but not validated, samples for IPA diagnosis. In fact, the GM median threshold from the TA sample in our study was higher than the 2.00 OD previously identified on the same sample in a cohort of 144 ICU patients as the best cutoff for CAPA screening according to modified AspICU criteria [6]. These authors also highlighted the NPV > 90% of the above-mentioned GM threshold for CAPA probable diagnosis. Moreover, our GM cutoff was closer to the studies on which our definition of possible CAPA was based (3.25 vs. 3.33); we also shared the same methodology, aside from the lack of physiological saline sweep [4,11]. Furthermore, we demonstrated the coexistence of positive cultures in 37% of cases, in agreement with clinical guidelines indicating a different percentage depending on the basal condition, set at 28% for critically ill non-neutropenic patients. A recent systematic *Aspergillus* spp. screening proposal using PCR and/or cultures described an overall concordance of 86% between sequentially collected TA and BAL samples. BAL confirmed all culture-positive TA samples [5]. These authors recommend minimally invasive screening by TA, followed by BAL performance in positive cases. The importance of bronchoscopy was recently highlighted in a large cohort of critically ill patients diagnosed with COVID-19 and ARDS, where GM testing had higher sensitivity from BAL (77% with 1 ODI cutoff) than from serum (19%) [13]. We measured serum BDG instead of serum GM. BDG fungal biomarker was present in a quarter of cases (none of which were diagnosed with candidemia), which would support the presence of budding invasive fungal disease. 

Second, we were able to analyze changes in GM values in the 19 cases during their ICU stay (Figure 1), which appear to have at least visual prognostic value. There was a trend to diminish or even normalize the GM index in patients who survived despite a high initial value, as well as in most patients with low values at the beginning, but mortality increased when it remained stable or tended to be higher in short-term follow-up. 

Third, the incidence and timing of CAPA-IP in the current study match with those described for CAPA in previous published series addressing the ICU population. Most of these real-life studies made limited use of bronchoscopy and BAL for CAPA definition and diagnosis, and are, instead, partly supported by TA [6,7,8,9,10]. Moreover, our subset presented the same mortality rate as was reported in other ICU population studies in which antifungal treatment was also implemented [6,10,13,15]. Curiously, we observed similar CAPA-IP mortality rates in an earlier stage of the process, which could be explained either by the sizeable percentage of TA samples included in previous studies or by high concordance between TA and BAL for GM [13], which were detected earlier following an active aspergillosis search approach. Of note, one of the key findings in this regard comes from a recently published French cohort [10], where a high mortality rate of 45.8% was observed in 24 (5%) patients with non-bronchoalveolar lavage or bronchial or TA positive for *Aspergillus* spp. culture and/or PCR, and a compatible clinical context of aspergillosis compared with non-CAPA. 

Finally, American and European guidelines strongly recommend early initiation of antifungal therapy in patients with highly suspected IPA during diagnostic evaluation [16,17,18]. Small prospective series suggest that poor outcomes might potentially be improved using antifungal therapies [9,15,19]. Subsequent larger cohorts showed that among secondary infections, only fungal coinfection was significantly associated with death. In the European cohort, 52% of patients who received antifungals were alive at ICU discharge, in contrast with only 10% of those who did not. Given that CAPA seems to develop later than IAPA (median 8 vs. 3 days), these authors proposed prophylaxis as a promising strategy in ICU COVID-19 patients [13]. Likewise, in the French MYCOVID study, although introducing a triazole did not modify mortality in patients with proven or probable CAPA, the authors also focused on the possible benefit of prophylaxis or pre-emptive strategies for high-risk patients, including those receiving dexamethasone combined with anti-IL6 [10]. Our results are similar to these studies, both in mortality rates in patients receiving antifungal therapy and in the lack of difference between triazole treatments. In our real-life approach, we started therapy as soon as the GM assay was positive on TA (pre-emptive strategy) and the median antifungal treatment time was almost half the 6–8 weeks recommended for well-defined IPA (the so-called “short course” in Figure 2), so this could pave the way for a new stewardship approach. 

Our study has significant limitations, firstly those inherent to single-center studies. Secondly, our study was developed across the six waves of the COVID-19 pandemic, with differing numbers of ICU-admitted patients (most of them requiring IMV), different hospital conditions, and a wide variety of empiric and targeted therapies applied, all of which could influence and explain the overall results. Finally, we did not obtain systematic matched samples from BAL following a positive GM result in AT, which could have served to underline the importance of this test, both for confirming diagnosis during the disease course and determining whether systemic antifungal treatment should be withheld. This explains the inclusion of BAL in the proposed algorithm. There was no unified criterion among physicians for ending antifungal treatment. 

## 5. Conclusions

To summarize, despite these limitations and pending external validation, we believe that our results add useful data to the currently available body of literature. To prevent COVID-19-associated progression of aspergillosis pulmonary disease, this new simpler concept of CAPA-IP, which highlights the role of TA sampling, could help provide active surveillance or diagnose an emerging process, and thus prompt implementation of a pre-emptive and shorter treatment strategy; this, in turn, could improve prognosis in these critically ill patients, specifically in certain centers and particular situations. 

## Figures and Tables

**Table 1:** Microbiological data and systemic antifungal therapy from patients fulfilling the CAPA-IP criteria.

Patients	TA GM Index at the Time of Clinical Deterioration	Serum BDG (pg/mL)	TA MALDI- TOF Identification	Score Value of MALDI- TOF	TA PCR *Aspergillus* spp. at the Time of Clinical Deterioration	Pre- PTZ	Number of Mycological Criteria	ANTIFUNGAL	THERAPY
								Type and Duration	ICU Outcome
1	0.98	-	-		-	yes	1	Anidulafungin 5d.>> Isavuconazole 10d.	Survival
2	0.74	<2.5	*A. terreus*	2.03	Positive Ct30	yes	3	Isavuconazole 23d.	Survival
3	0.80	-	-		-	no	1	NO one	NON-survival
4	5.28	-	-		-	no	1	Isavuconazole 21d.	Survival
5	2.74	<2.5	-		-	no	1	Isavuconazole 28d.	Survival
6	5.83	<2.5	-		-	no	1	Voriconazole 11d.	Survival
7	9.99	65.17	-		-	no	2	NO one	NON-survival
8	1.54	-	-		-	yes	1	Isavuconazole 12d.	Survival
9	5.79	12.00	-		-	no	2	Isavuconazole 8d.	NON-survival
10	7.16	-	*A. fumigatus*	1.94	-	yes	2	Voriconazole 21d.	Survival
11	0.96	-	*A. terreus*	2.02	Positive Ct32	no	3	Voriconazole 3d.	NON-survival
12	5.30	<2.5	*A. terreus*	2.21	Positive Ct30	no	3	Isavuconazole 9d.>> Amphotericin B 1d.>> Posaconazole + Caspofungin 14d.	NON-survival
13	0.90	32.16	-		-	no	2	Isavuconazole 5d.	NON-survival
14	2.07	-	-		-	no	1	Voriconazole 7d.>> Isavuconazole + Anidulafungin 21d.	NON-survival
15	1.83	<2.5	*A. flavus*	1.97	-	no	2	Isavuconazole 14d.	NON-survival
16	0.6	<2.5	-		Positive Ct32	no	2	Isavuconazole 23d.>> Amphotericin B 6d.	NON-survival
17	0.6	<2.5	-		-	no	1	Isavuconazole 21d.	NON-survival
18	5.7	25.00	*A. fumigatus*	1.89	-	yes	3	Isavuconazole 21d.	Survival
19	0.6	15.78	*A. fumigatus*	2.23	Positive Ct31	no	4	Voriconazole 12d.	Survival

Note: CAPA-IP: CAPA in progress; GM: galactomannan; BDG: 1.3-β-d-glucan; TA: tracheal aspirate; PCR: polymerase chain reaction assay; Ct: Cycle threshold; pre-PTZ: previous treatment with piperacillin/tazobactam; ICU: intensive care unit; d.: days; >>: salvage treatment.

## Data Availability

The data presented in the manuscript have not been made available but can be shared upon request.
